# RhoBTB1 interacts with ROCKs and inhibits invasion

**DOI:** 10.1042/BCJ20190203

**Published:** 2019-09-13

**Authors:** Raquel B. Haga, Ritu Garg, Francesca Collu, Bárbara Borda D'Agua, Sofia T. Menéndez, Audrey Colomba, Franca Fraternali, Anne J. Ridley

**Affiliations:** 1 Randall Centre of Cell and Molecular Biophysics, King's College London, New Hunt's House, Guy's Campus, London SE1 1UL, U.K.; 2 School of Cellular and Molecular Medicine, University of Bristol, Biomedical Sciences Building, University Walk, Bristol BS8 1TD, U.K.

**Keywords:** cell invasion, Cullin3, phosphorylation, Rho GTPases, Rho-kinases, RhoBTB

## Abstract

RhoBTB1 is an atypical Rho GTPase with two BTB domains in addition to its Rho domain. Although most Rho GTPases regulate actin cytoskeletal dynamics, RhoBTB1 is not known to affect cell shape or motility. We report that RhoBTB1 depletion increases prostate cancer cell invasion and induces elongation in Matrigel, a phenotype similar to that induced by depletion of ROCK1 and ROCK2. We demonstrate that RhoBTB1 associates with ROCK1 and ROCK2 and its association with ROCK1 is via its Rho domain. The Rho domain binds to the coiled-coil region of ROCK1 close to its kinase domain. We identify two amino acids within the Rho domain that alter RhoBTB1 association with ROCK1. RhoBTB1 is a substrate for ROCK1, and mutation of putative phosphorylation sites reduces its association with Cullin3, a scaffold for ubiquitin ligases. We propose that RhoBTB1 suppresses cancer cell invasion through interacting with ROCKs, which in turn regulate its association with Cullin3. Via Cullin3, RhoBTB1 has the potential to affect protein degradation.

## Introduction

**Figure 1.**
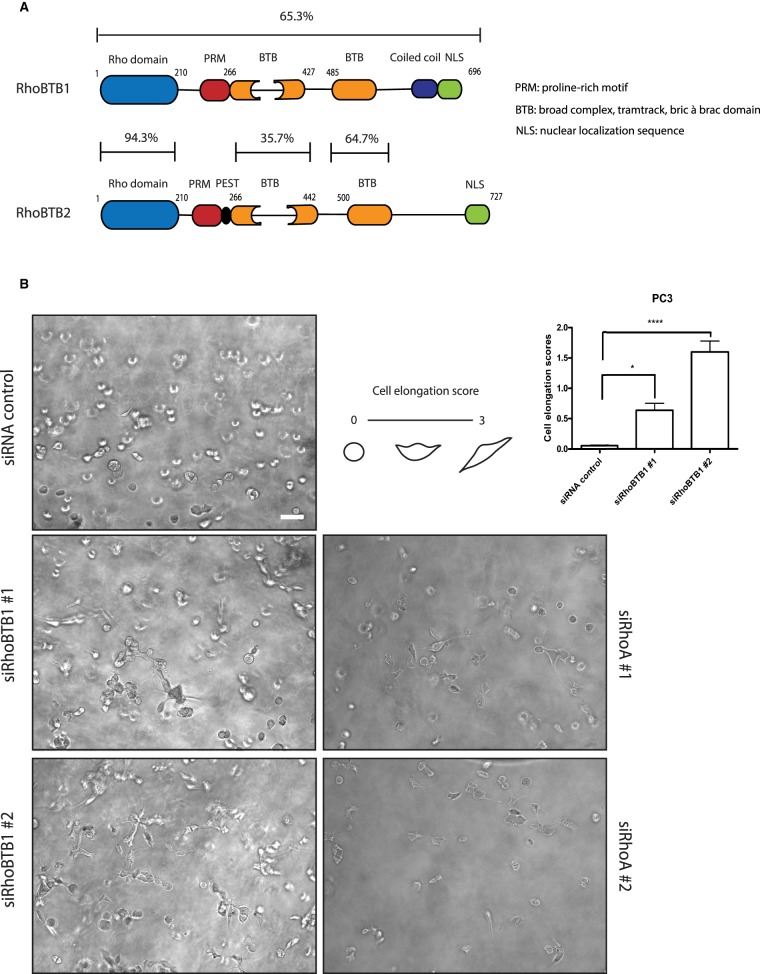
RhoBTB1 depletion affects the morphology of PC3 cells. (**A**) Diagram showing RhoBTB1 and RhoBTB2 domains (amino acid numbering is for human proteins). Full-length human RhoBTB1 is 65.3% identical with human RhoBTB2. The Rho domain shows 94.3% identity while the BTB domains show 35.7% and 64.7% identity, respectively. (**B**) PC3 cells were transfected with siRNA oligos targeting RhoBTB1, RhoA or siRNA control. After 48 h, cells were embedded in Matrigel. Phase-contrast images were taken after 24 h. Images are representative of three independent experiments. Cells were scored based on their elongation: 0 = rounded morphology and 3 = elongated morphology. The graph shows quantification of cell elongation scores. Ten different fields from each condition were analyzed per experiment. Values represent mean ± SEM. * *P* < 0.05, **** *P* < 0.0001, compared with siRNA control, determined by one-way ANOVA analysis of variance followed by a Dunnett's multiple comparison. Scale bar = 100 µm.

Members of the Rho GTPase family are known to regulate cytoskeletal dynamics and cell adhesion. The classical members of this family are molecular switches cycling between an active GTP-bound form and an inactive GDP-bound form. The ratio of GTP-bound form/GDP-bound form is regulated by guanine nucleotide exchange factors (GEFs), GTPase-activating proteins (GAPs) and guanine nucleotide dissociation inhibitors (GDIs) [1]. However, several Rho GTPase family members have been shown to be constitutively GTP-bound, including Rnd proteins and RhoH [2]. RhoBTB1 and RhoBTB2 are atypical members of the family that are predicted not to cycle between a GTP-bound form and a GDP-bound form, based on their amino acid sequences [3]. These proteins are larger than classical Rho GTPases due to the presence of extra domains in addition to the conserved GTP-binding Rho domain [4]. The Rho domain is followed by a proline-rich motif (PRM), a tandem of 2 broad-complex, tramtrack, bric à brac (BTB) domains, and a conserved C-terminal region (Figure 1A). The BTB domains in RhoBTB proteins have been shown to mediate homo- and heterodimerization and interaction with Cullin3 [5], a scaffold protein in the ubiquitin ligase complexes [6].

Little is known of the functions or regulation of RhoBTB1 and RhoBTB2, and there is no evidence so far that they have any effects on the actin cytoskeleton or cell migration. RhoBTB1 and RhoBTB2 were reported to localize in part to vesicles [7], but whether they affect vesicle trafficking is not known [4]. They are unlikely to be regulated by GAPs or GEFs, and, in contrast with most Rho GTPases, do not have a C-terminal CAAX box and hence are not prenylated [4,8]. Whether they are regulated by post-translational modifications found on other Rho GTPases, such as phosphorylation, SUMOylation and ubiquitination [9], has not been explored.

Here we show for the first time that a decrease in RhoBTB1 expression leads to changes in cell morphology and increased invasion of cancer cells. We also report that RhoBTB1 associates with ROCK1 through its Rho domain and that this leads to RhoBTB1 phosphorylation. We propose that RhoBTB1/ROCK complexes could act together to regulate cancer cell morphology and invasion.

## Experimental

### Cell lines and reagents

COS7, HeLa and MDA-MB-231 cells were grown in DMEM supplemented with 10% FCS, 1% pyruvate. PC3 cells were grown in RPMI supplemented with 10% FCS. All media contained 100 µg/ml streptomycin and 100 U/ml penicillin. The following antibodies were used: myc tag (A-14, sc-789, or 9E10, sc-40; Santa Cruz Biotechnology), GFP (FL) (sc-8334; Santa Cruz Biotechnology), Cullin3 (611848; BD), ROCK1 (611136; BD), ROCK2 (610623; BD), HA tag (3F10; Sigma–Aldrich), pMLC2 (Thr18/Ser19) (#3674, Cell Signaling), MLC2 (#3672, Cell Signaling), RhoA 67B9 (#2117, Cell Signaling), GAPDH (MAB374, Merck Millipore). Secondary horseradish peroxidase (HRP)-labelled antibodies were from GE Healthcare (anti-mouse, anti-rat and anti-rabbit). Complete protease inhibitor cocktail and PhosphoStop were from Roche. Active recombinant human GST-ROCK1 (17–535, # R10–11G) was from SignalChem. MLN4924 was from BostonBiochem (R&D Systems). H1152 Rho kinase inhibitor was from Calbiochem.

### Expression vectors and site-directed mutagenesis

Full-length and deletion mutants of human ROCK1, pCAG-myc-ROCK1, ROCK1^1–1080^, ROCK1^1–727^, were a gift from Shuh Narumiya (Kyoto University, Japan) [10]. pCAG-myc-ROCK1^1–727^ (K105A), ROCK1^1–540^, ROCK1^1–420^, ROCK^375–727^ and ROCK1^1096–1354^ were previously described [11]. Full-length rat pXJ-HA-ROCK2 was a gift from Ed Manser (Institute of Molecular and Cell Biology, Singapore). pCAG-myc-ROCK2^1–436^ was generated from full-length bovine pCAG-myc-ROCK2 (kindly provided by Erik Sahai, Crick Institute, London, U.K.). The region encoding ROCK2^1–436^ was amplified by PCR using BamH1 forward primer (CGGATCCATGAGCCGGCCCCCGCCGACGGGG) and EcoR1 reverse primer (CGGAATTCTCATGATTGAATTGAGTCATTTT). The PCR product was cloned into the BamH1/EcoR1 sites of pCAG. The construct was verified by restriction enzyme digestion and DNA sequencing (MWG-Eurofins, U.K.).

Human pRK5-myc-RhoBTB1 was kindly provided by Pontus Aspenström (Karolinska Institute, Stockholm, Sweden) [7] and GFP-RhoA^G14V^ by Ferran Valderrama (St George's, University of London) [12]. RhoBTB1 and RhoBTB1^1–210^ cDNA were subcloned into pEGFP-C1 and CB6-GFP, respectively. The expression vectors encoding pRK5-myc-RhoBTB1 and pEGFP-RhoBTB1 were mutagenized using QuikChange II Site-Directed Mutagenesis Kit (Agilent Technologies). The nucleotide changes were verified by DNA sequencing (MWG-Biotech).

### DNA and siRNA transfection

COS7 cells and HeLa cells were transfected with different plasmids using Lipofectamine 2000 (Invitrogen) in complete medium without antibiotics. The medium was changed to complete medium 6 h after transfection. After 24 h of transfection, cells were lysed or fixed for immunostaining.

All siRNAs were from Eurogentec and Dharmacon (GE Healthcare): siRNA control (UUCUCCGAACGUGUCACGU), siRhoBTB1 #1 (AAAUGAAGCUGCCUGUUUA), siRhoBTB1 #2 (GAACUUGGCUUACCAUACU), siRhoBTB1 #4 (GAACACCCGUUAUCCUUGU), siRhoA #1 (AUGGAAAGCAGGUAGAGUU), siRhoA #2 (GAACUAUGUGGCAGAUAUC), siROCK1 #2 (GAAGAAACAUUCCCUAUUC), siROCK1 #3 (GAGAUGACAAGUCAAUUA), siROCK2 #5 (GCAAAUCUGUUAAUACUCG) and siROCK2 #9 (CAAACUUGGUAAAGAAUUG). Cells were transfected with 50 nM siRNA using Oligofectamine (Invitrogen) in complete medium without antibiotics. The medium was changed to complete medium 6 h after transfection. After 72 h, cells were lysed in lysis buffer (50 mM Tris–HCl pH 8, 150 mM NaCl, 5 mM EGTA, 1% Triton X-100, supplemented with complete protease inhibitor cocktail (Roche), 25 mM NaF and 2 mM Na_3_VO_4_) and samples were analyzed by immunoblotting.

### qPCR

Extraction of mRNA from cells using the RNeasy Mini Kit (following the manufacturer's instructions) was performed 72 h after siRNA transfection. qPCR was carried out using Brilliant III Ultra-Fast SYBR® Green QRT-PCR Master Mix. Each condition was carried out in triplicate and GAPDH was used as a reference gene (internal control). The assay was carried out using the Stratagene MX3500PTM sequence detection system and the amplification cycles were carried out following the manufacturer's instructions. For the analysis of raw data, the MxPro software was used. Quantification of the amplified cDNA was achieved by comparing the number of amplification cycles (Ct) after which the fluorescent signal crossed a threshold level. The following formula was applied to quantify the results:

$$\begin{array}{rl} 2-\Delta \Delta \text{CT} & =[((\text{Ct gene of interest}–\text{Ct internal control})\text{sample A}\\ & \;–(\text{Ct gene of interest}–\text{Ct internal control})\text{sample B})] \end{array}$$

### Immunoprecipitation and immunoblotting

COS7 cells transfected with plasmids were lysed in IP lysis buffer (1% Triton X-100, 20 mM Tris pH 8, 130 mM NaCl, 1 mM DTT, supplemented with protease inhibitor cocktail complete (Roche) and PhosphoStop (Roche)). After clarifying the samples, lysates were incubated with myc-trap or GFP-trap beads (ChromoTek). The immunoprecipitates were washed with IP lysis buffer, and the proteins were eluted with NuPAGE LDS Sample Buffer (Invitrogen) containing 5% β-mercaptoethanol. Samples were used in other assays or analyzed by immunoblotting. Lysates were resolved on SDS-polyacrylamide gels and transferred to nitrocellulose membranes. Membranes were blocked using 5% dried milk in Tris-buffered saline with 0.1% Tween-20 (TBS-T) and incubated with primary antibody overnight at 4°C. Membranes were washed three times with TBS-T and incubated for 1 h at room temperature with appropriated HRP-labelled secondary antibody. Enhanced chemiluminescence (ECL; GE Healthcare) was used as a detection reagent.

### *In vitro* kinase assay

Myc-tagged ROCK1 and GFP-RhoBTB1 or myc-RhoBTB1 were expressed in COS7 cells. After 24 h, cells were lysed in IP buffer and proteins were immunoprecipitated with myc-trap or GFP-trap beads (ChromoTek). Samples were washed with high salt buffer (1% Triton X-100, 20 mM Tris pH 8, 250 mM NaCl, 1 mM DTT). Beads were combined depending on the conditions of the experiment and then pelleted by centrifugation. Beads were resuspended in kinase mix (40 mM MgCl_2_, 4 mM MnCl_2_, 60 mM Tris pH 7.4, 30 µM ATP, 0.1 µCi/µl [^32^P] γATP). Recombinant GST-ROCK1^17–535^ was added in some of the conditions. Samples were incubated at 30°C for 30 min. Proteins were eluted with NuPAGE LDS Sample Buffer (Invitrogen) containing 5% β-mercaptoethanol. Samples were resolved in 4–12% SDS-polyacrylamide gels and fixed in 8.75% acetic acid, 25% ethanol for 30 min. Gels were dried for 2 h at 70°C and imaged by autoradiography.

### Protein phosphorylation analysis by Pro-Q diamond staining

COS7 cells transfected with plasmids were lysed after 24 h with IP buffer, and myc-RhoBTB1 was immunoprecipitated with anti-myc antibodies. Samples were resolved in an SDS-polyacrylamide gel and stained using Pro-Q Diamond phosphoprotein gel stain kit (Molecular Probes, Invitrogen), following the manufacturer's instructions. Bands were visualized using a UV-transilluminator. Samples were also analyzed by immunoblotting to determine total amount of myc-RhoBTB1.

### Immunostaining

HeLa cells transfected with plasmids were fixed with 4% paraformaldehyde for 15 min, permeabilized with 0.1% Triton X-100 in PBS for 5 min and blocked with 3% bovine serum albumin (BSA) in PBS for 30 min. Cells were incubated with mouse anti-myc (9E10) overnight at 4°C followed by anti-mouse Alexa Fluor 546 antibody for 45 min. DAPI was used for nuclear staining. Confocal images were acquired with an inverted confocal microscope (LSM510; Carl Zeiss) using a 63× (1.4 NA) objective.

### 3D morphology assay

The analysis of cell morphology was carried out as previously described [13]. Briefly, a 96-well plate was coated with 40 µl of a 7.5 mg/ml Matrigel solution. Around 5 × 10^5^ cells transfected with siRNA oligonucleotides 48 h before the assay were mixed with 7 mg/ml Matrigel solution and transferred into one of the wells in the Matrigel-coated plate. The plate was incubated at 37°C. After 2 h, 100 µl of appropriated medium without FCS was added to each well. Cells were incubated at 37°C for 24 h. Images were acquired using a Nikon TE2000-E microscope with a Plan Fluor 10× objective (Nikon) and a Hamamatsu Orca-ER digital camera.

### Transwell invasion assay

PC3 cells in RPMI containing 0.1% FCS were added to the top chamber of Matrigel-coated invasion chambers (8 µm pore diameter; BD Biosciences), and 1% FCS/RPMI was added to the lower chamber as an attractant. After 24 h, cells on the bottom of the Matrigel-coated transwells were fixed with 70% ethanol and stained with 0.2% crystal violet. Random images from independent experiments were acquired using a Nikon Eclipse TS100 microscope with a 10x objective and cells were counted using ImageJ (Plugin: cell counter).

### Statistical analysis

Results were analyzed by one-way analysis of variance (ANOVA) followed by Dunnett's multiple comparisons test. Statistical significance is indicated by * *P* < 0.05, ** *P* < 0.01, *** *P* < 0.001, **** *P* < 0.0001 as compared with the control.

## Results

### Depletion of RhoBTB1 affects the morphology of PC3 cells in 3D matrices

Ectopic expression of RhoBTB1 and RhoBTB2 has been reported to have only a minor effect on actin filament organization in primary aortic endothelial cells [7]. However, the experiments were conducted with cells on glass in 2D, without the presence of any added extracellular matrix component. We have established conditions to study the effects of different genes on the morphology of cells embedded in a 3D Matrigel matrix [13]. For example, PC3 prostate cancer cells spread on 2D surfaces, whereas they have a rounded morphology in 3D Matrigel (Supplementary Figure S1A). In Matrigel, they extend protrusions in response to HGF stimulation or depletion of RhoA or the RNA-binding protein LARP4 [13,14]. We identified RhoBTB1, but not RhoBTB2, as a potential hit in a 3D Matrigel RNAi morphology screen using PC3 cells (Supplementary Figure S1B) [13]. RhoBTB1 and RhoBTB2 have similar domain structures, but they are only 65.3% identical at the amino acid level (Clustal Omega, [15]) (Figure 1A), and hence are likely to have different functions in cells.

The RNAi morphology screen used pools of four siRNAs [16], so to identify single siRNAs that effectively knocked down RhoBTB1, PC3 cells were transfected with three of the four RhoBTB1 siRNAs present in the siRNA pool, which all depleted RhoBTB1 mRNA levels (Supplementary Figure S1C). Available antibodies to RhoBTB1 did not reliably detect endogenous RhoBTB1 on western blots (data not shown). Subsequently, two of these siRNAs were selected for further experiments and tested for their ability to alter PC3 cell morphology in Matrigel. Both siRNAs stimulated the extension of protrusions by PC3 cells, similar to the effect of the RhoBTB1 siRNA pool (Supplementary Figure S1B) and to RhoA depletion (Figure 1B and Supplementary Figure S1C). Cell morphology was assessed using a cell elongation score in which rounded cells were assigned a score of 0 and elongated cells scored 1 to 3 depending on the extent of elongation [14] (Figure 1B). Depletion of RhoBTB1 resulted in the elongation of PC3 cells (Figure 1B). Most control siRNA-transfected cells remained rounded in Matrigel, whereas cells transfected with the two siRNAs targeting RhoBTB1 became more elongated, with a cell elongation score between 0.5 and 1.5.

When PC3 cells were grown in 2D, depletion of RhoBTB1 led to an increase in cell area and cell perimeter but did not consistently alter circularity (which is the inverse of cell elongation; https://imagej.nih.gov/ij/plugins/circularity.html) (Supplementary Figure S1D). These results indicate that the effects of RhoBTB1 on cell shape depend on the extracellular environment.

### Depletion of RhoBTB1 increases cell invasion

**Figure 2.**
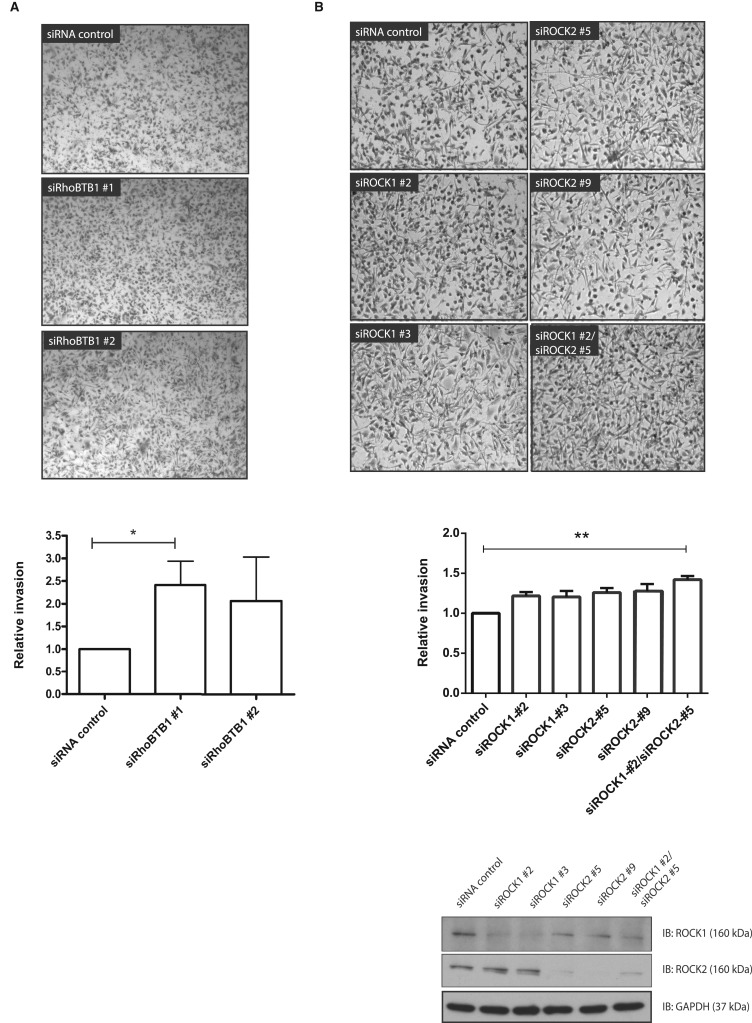
RhoBTB1 depletion increases invasion of PC3 cells. PC3 cells were transfected with siRNA control or siRNA oligos targeting RhoBTB1 (**A**) or ROCK1 and/or ROCK2 (**B**). After 48 h, cells were seeded on the top chamber of Matrigel-coated transwells in 0.1% FCS/RPMI. 1% FCS/RPMI was added to the bottom chamber to create a gradient. After 24 h, cells were fixed with 70% ethanol and stained with 0.2% crystal violet. Images are representative of four (**A**) or three (**B**) independent experiments. Graphs show relative invasion compared with siRNA control. Five different fields of each condition were analyzed per experiment. Values represent mean ± SEM. * *P* < 0.05, ** *P* < 0.01 compared with siRNA control, determined by one-way ANOVA analysis of variance followed by a Dunnett's multiple comparison.

We previously observed that RhoA-depleted cells have an elongated morphology and that this leads to increased invasion [17]. We, therefore, tested the effects of RhoBTB1 depletion on cell invasion. Depletion of RhoBTB1 increased PC3 cell invasion through Matrigel-coated transwells by two-fold (Figure 2A), similar to the reported effect of RhoA depletion [17]. RhoA acts through its two targets ROCK1 or ROCK2 [18], and consistent with our previous results [17], ROCK1 or ROCK2 depletion also increased PC3 cell invasion (Figure 2B). These results suggest a phenotypic link between RhoBTB1 and Rho/ROCK signaling. Although RhoBTBs can associate with Cullin3, a known E3 ubiquitin ligase scaffold involved in RhoA degradation [19], depletion of RhoBTB1 did not affect RhoA protein levels in PC3 cells (Supplementary Figure S1E).

### ROCK1 associates with RhoBTB1 via its HR1-like region

**Figure 3.**
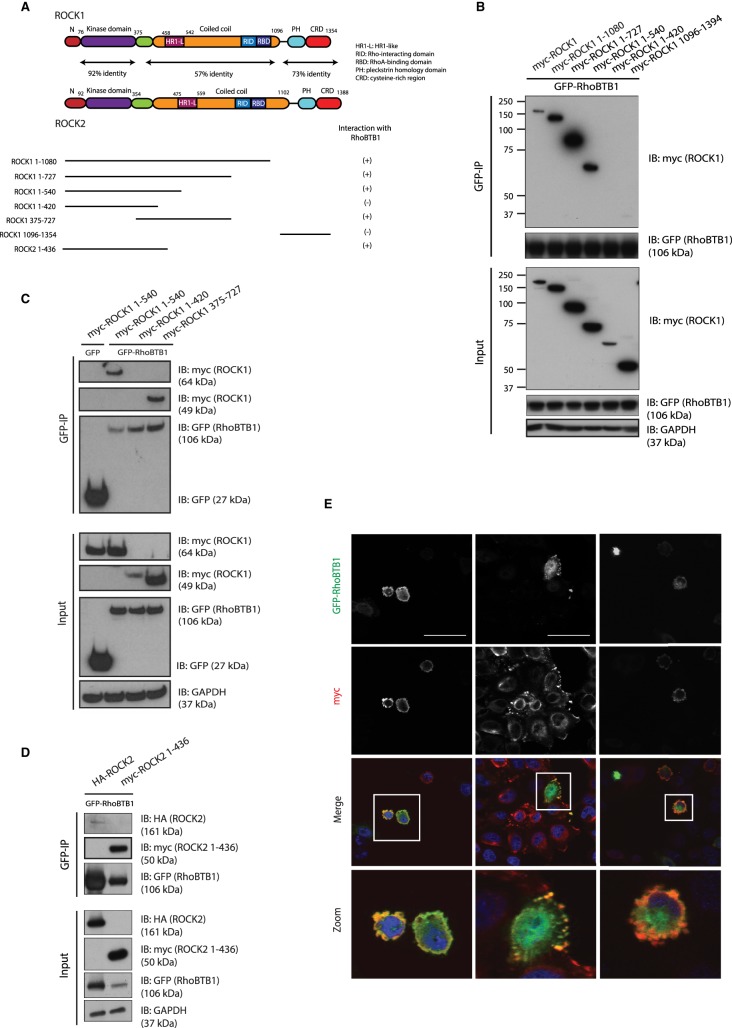
ROCK1 interacts with RhoBTB1 via its HR1-like domain. (**A**) Diagram showing ROCK1 and ROCK2 deletion mutants. (**B**–**D**) COS7 cells were transfected with pEGFP or vector encoding GFP-RhoBTB1 and the indicated myc-epitope tagged ROCK1 and ROCK2 constructs or HA-ROCK2. After 24 h, cells were lysed and incubated with GFP-binding protein-coupled to agarose (GFP-trap). Total lysates (input) and immunoprecipitates (GFP-IP) were probed to show levels of myc-ROCK1 deletion mutants, myc-ROCK2^1–436^, HA-ROCK2 and GFP-RhoBTB1. GAPDH is used as a loading control. (**E**) HeLa cells were transfected with vectors encoding GFP-RhoBTB1, myc-ROCK1^1–420^, myc-ROCK1^1–540^ and myc-ROCK1^375–727^. After 24 h, cells were fixed and stained with anti-myc epitope antibody and for nuclei (DAPI). Scale bar = 50 µm.

To investigate whether there is a biochemical link between RhoBTB1 and Rho/ROCK signalling, we tested RhoBTB1 association with ROCKs, using a range of ROCK1 and ROCK2 deletion mutants (Figure 3A). Full-length RhoBTB1 co-immunoprecipitated with ROCK1 (Figure 3B). To determine which region of ROCK1 is involved in its association with RhoBTB1, the deletion mutants of ROCK1 were co-expressed with full-length RhoBTB1 in COS7 cells. RhoBTB1 associated with full-length ROCK1, ROCK1^1–1080^, ROCK1^1–727^, ROCK1^375–727^ and ROCK1^1–540^, but much less with ROCK1^1–420^ and not detectably with ROCK1^1096–1394^ (Figure 3B,C). These data together indicate that the region of ROCK1 between amino acids 420 and 540 is required for optimal association with RhoBTB1 (Figure 3A).

To investigate RhoBTB1 association with ROCK2, full-length ROCK2 and a construct encoding the kinase domain together with N- and C-terminal regions (ROCK2^1–436^; similar to ROCK1^1–420^; Figure 3A,D) were co-expressed with full-length RhoBTB1. ROCK2^1–436^ co-immunoprecipitated with RhoBTB1 whereas ROCK1^1–420^ was not detected, suggesting that this region of ROCK2 may bind more tightly with RhoBTB1 than the equivalent region of ROCK1 (Figure 3D).

Interestingly, the region in ROCK1 between amino acids 420 and 540 contains an HR1-like region (Figure 3A) [20]. HR1 domains are found in a variety of Rho targets, and they are important for binding of RhoA to PKNs and rhotekin [21,22]. This region of ROCK1 was suggested to contribute to RhoA binding in addition to the Rho-binding domain [20]. Consistent with this, we observed that constitutively active RhoA^G14V^ co-immunoprecipitated with ROCK1^1–727^ similarly to RhoBTB1, but also unexpectedly co-immunoprecipitated with ROCK1^1–420^ (which lacks the HR1-like domain; Supplementary Figure S2A), suggesting that there is another as-yet-unidentified region within the N-terminal region of ROCK1 that associates with RhoA.

Given that RhoBTB1 and ROCK1 co-immunoprecipitated from cells, we tested whether they co-localize. Vectors encoding GFP-RhoBTB1 were co-transfected with different ROCK1 deletion mutants into HeLa cells (Figure 3E). ROCK1^1–420^ and ROCK1^1–540^ induced cell contraction, consistent with results previously observed with active ROCK1 [11]. RhoBTB1 and ROCK1^1–540^ co-localized around the cell periphery, whereas this co-localization was not observed in cells co-expressing RhoBTB1 and ROCK^11–420^ (Figure 3E). ROCK1^375–727^, which associates with RhoBTB1 but lacks the kinase domain and hence does not alter cell shape but associates with RhoBTB1 (Figure 3A), co-localized frequently with RhoBTB1 to punctate and/or ruffle-like structures around the cell periphery (Figure 3E). These results together indicate that RhoBTB1 and ROCK1 partially co-localize in cells when they are able to associate with each other.

### RhoBTB1 interacts with ROCK1 through its Rho domain

**Figure 4.**
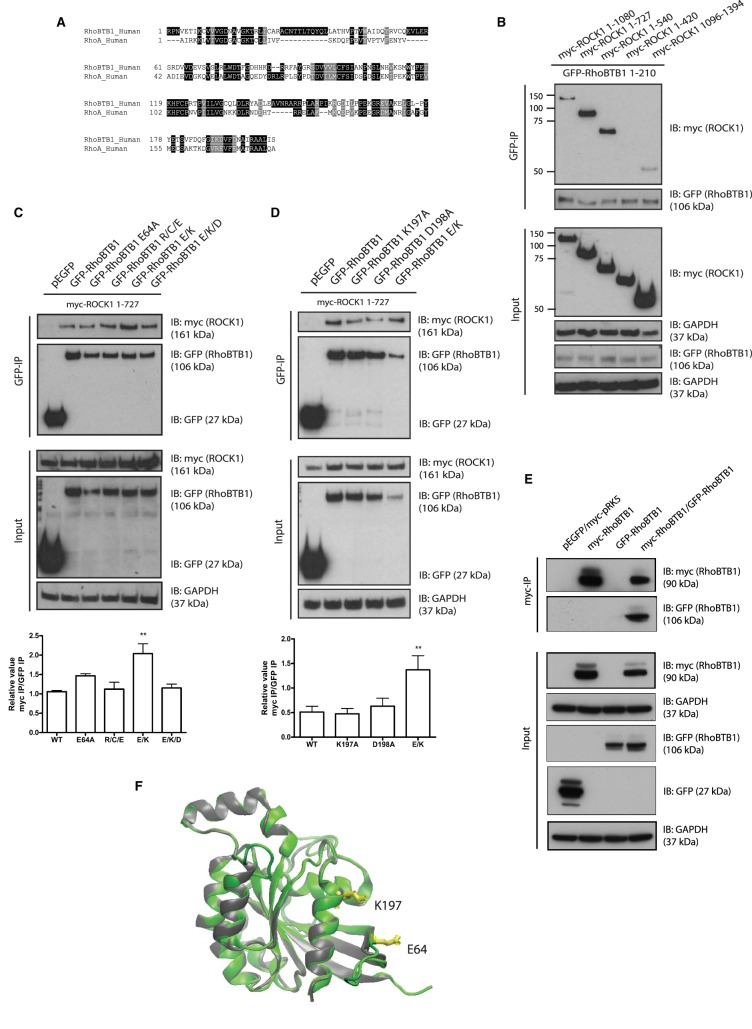
RhoBTB1 associates with ROCK1 through its Rho domain and mutation of E64 and K197 on RhoBTB1 affects this interaction. (**A**) Alignment of Rho domain of RhoBTB1 and RhoA. (B-E) COS7 cells were transfected with (**B**) a vector encoding GFP-RhoBTB1^1–210^ and the indicated myc-epitope tagged ROCK1 constructs, (**C**) empty pEGFP and vectors encoding myc-ROCK1^1–727^, GFP-RhoBTB1, GFP-RhoBTB1 R/C/E (R60A/C62A/E64A), GFP-RhoBTB1^E64A^, GFP-RhoBTB1 E/K (E64A/K197A) and GFP-RhoBTB1 E/K/D (E64A/K197A/D198A), (**D**) empty pEGFP and vectors encoding myc-ROCK1^1–727^, GFP-RhoBTB1, GFP-RhoBTB1^K197A^, GFP-RhoBTB1^D198A^, GFP-RhoBTB1 E/K (E64A/K197A) and (**E**) empty pEGFP, empty myc-pRK5 and vectors encoding myc-RhoBTB1 and GFP-RhoBTB1. After 24 h, cells were lysed and incubated with GFP-binding protein-coupled to agarose (GFP-trap) (**B**–**D**) or anti-myc-agarose beads (**E**). Total lysates (input) and immunoprecipitates were probed to show levels of myc-ROCK1 mutants, myc-RhoBTB1, GFP-RhoBTB1, GFP-RhoBTB1^1–210^ and GFP-RhoBTB1 mutants. GAPDH is used as a loading control. (**C** and **D**) The graph shows the quantification of the band density from three (**C**) or five (**D**) independent experiments. Band density of myc-ROCK1^1–727^ (myc-IP) was normalized by GFP-RhoBTB1 constructs (GFP IP). All values were normalized to GFP-RhoBTB1 WT condition. Values represent mean ± SEM. ** *P* < 0.01, compared with GFP-RhoBTB1 WT, determined by one-way ANOVA analysis of variance followed by a Dunnett's multiple comparison. (**F**) A computational model of the Rho domain of RhoBTB1 (green) modeled by using as a template the crystal structure of RhoA (gray; PDB number 1S1C) in complex with Rho binding domain of ROCK1. RhoBTB1 amino acids E64 and K197 are highlighted in yellow.

Since HR1 domains interact with RhoA, and the Rho domain of RhoBTB1 is the only domain that is conserved between RhoA and RhoBTB1 (31.5% amino acid identity; Figure 4A), we tested whether the RhoBTB1 Rho domain (RhoBTB1^1–210^; Figure 1A) associated with ROCK1. RhoBTB1^1–210^ co-immunoprecipitated with ROCK1^1–1080^, ROCK1^1–727^ and ROCK1^1–540^, but not with ROCK1^1–420^, indicating that the Rho domain is sufficient for complex formation between RhoBTB1 and ROCK1 (Figure 4B).

To investigate further the interaction between the Rho domain of RhoBTB1 and the HR1-like domain of ROCK1, a series of mutations were introduced into the Rho domain of RhoBTB1 (Figure 4C,D). Three of the mutations are in a 10-amino acid sequence that is unique to RhoBTBs, which is inserted just after the effector region (R60, C62 and E64; Supplementary Figure S2B). The other two are in a region near the C-terminus of the Rho domain, which is the last relatively well-conserved region across Rho GTPases (C-terminal α-helix A5), but only RhoBTB1 and RhoBTB2 have the consecutive residues K197 and D198 (Supplementary Figure S2B). The amino acids in the unique RhoBTB1 insert did not have any effect on the association of RhoBTB1 with ROCK1^1–727^, either individually or when all mutated together, arguing that this region does not confer specificity on complex formation between RhoBTB1 with ROCK1 (Figure 4C). Similarly, neither the K197A nor the D198A mutation altered ROCK1 association (Figure 4D). Surprisingly, the double RhoBTB1^E64A/K197A^ mutation enhanced the association of ROCK1 with RhoBTB1 (Figure 4C,D). These two residues are conserved in RhoBTB1 across multiple species (Supplementary Figure S2C), suggesting they contribute to RhoBTB1 function. We found that RhoBTB1 homodimerizes (Figure 4E), as previously reported for RhoBTB2 and RhoBTB3, mediated by their BTB domains (Supplementary Figure S3A) [5]. Interestingly, a structural model of the Rho domain of RhoBTB1 based on the RhoA structure (PDB 1S1C) indicates that E64 and K197 are on the same surface of the protein (Figure 4F). It is possible that, in the RhoBTB1 dimer, this surface forms a dimer interface between the two Rho domains, thereby partially occluding ROCK1 interaction. Mutation of these two residues would prevent this inhibitory effect and open up the Rho domain so that it can interact more strongly with ROCK1.

### ROCK1 phosphorylates RhoBTB1 and this phosphorylation alters the interaction of RhoBTB1 and Cullin3

**Figure 5.**
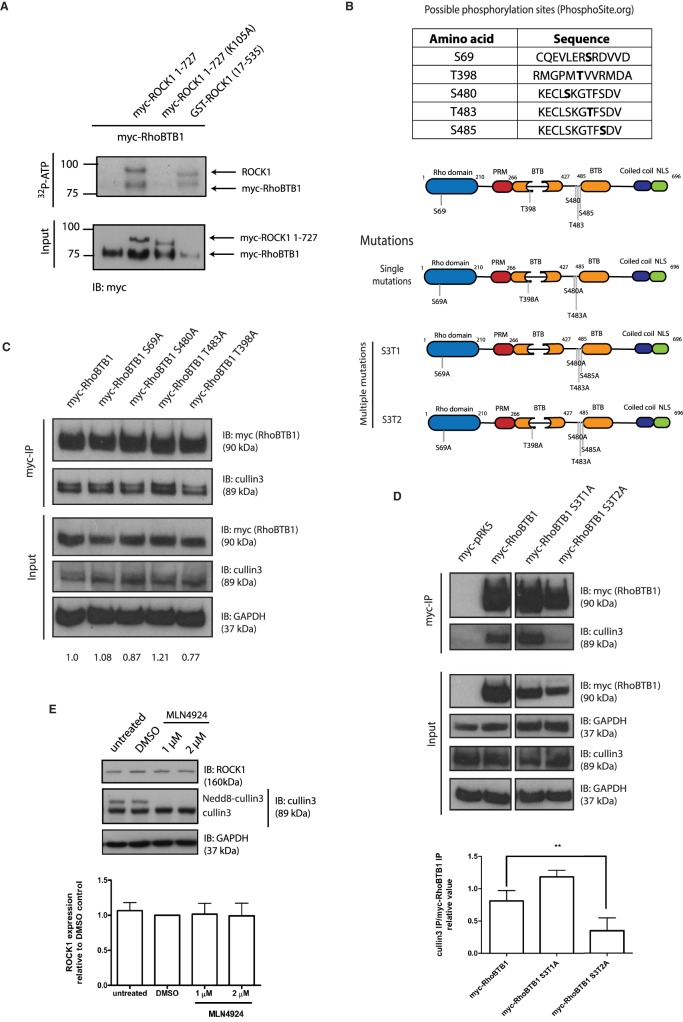
ROCK1 phosphorylates RhoBTB1 and this phosphorylation alters the association of RhoBTB1 and Cullin3. (**A**) COS7 cells were transfected with vectors encoding myc-RhoBTB1, myc-ROCK1^1–727^ or myc-ROCK1^1–727^ (K105A). After 24 h, cells were lysed and incubated with anti-myc-agarose beads. Immunoprecipitated lysates were combined as indicated (myc-RhoBTB1 alone, myc-RhoBTB1 and myc-ROCK1^1–727^, myc-RhoBTB1 and myc-ROCK1^1–727^ (K105A) (kinase-dead), and myc-RhoBTB1 and recombinant GST-ROCK1 17–535. Samples were incubated in a kinase buffer containing ^32^P-ATP for 30 min and then resolved in a 4–12% SDS-polyacrylamide gel. Bands show the phosphorylated proteins. Levels of myc-RhoBTB1, myc-ROCK1^1–727^ and myc-ROCK1^1–727^ (K105A) were analyzed in the total lysate (Input). (**B**) Table with sequence of potential phosphorylation sites on RhoBTB1 (PhosphoSite) and diagram showing the mutations on the RhoBTB1 sequence. (**C** and **D**) COS7 cells were transfected with (**C**) vectors encoding myc-tagged RhoBTB1, RhoBTB1^S69A^, RhoBTB1^S480A^, RhoBTB1^T483A^ and RhoBTB1^T398A^ and (**D**) empty pRK5-myc or vectors encoding myc-tagged RhoBTB1, RhoBTB1^S3T1A^ and RhoBTB1^S3T2A^. After 24 h, cells were lysed and incubated with anti-myc-agarose beads. Total lysates (input) and immunoprecipitates (myc-IP) were probed to show levels of (**C**) myc-RhoBTB1, RhoBTB1^S69A^, RhoBTB1^S480A^, RhoBTB1^T483A^ and RhoBTB1^T398A^ and Cullin3, and (**D**) myc-RhoBTB1, RhoBTB1^S3T1A,^ RhoBTB1^S3T2A^ and Cullin3. The interaction between myc-RhoBTB1 and Cullin3; and myc-RhoBTB1 mutants and Cullin3, is shown in the immunoprecipitates (myc-IP). GAPDH was used as a loading control. (**D**) The graph shows the quantification of the band density from four independent experiments. Band density of Cullin3 (myc-IP) was normalized by myc-RhoBTB1 (myc-IP). All values were normalized to myc-RhoBTB1 condition. Values represent mean ± SEM. ** *P* < 0.01, compared with myc-RhoBTB1 WT, determined by one-way ANOVA analysis of variance followed by a Dunnett's multiple comparison. (**E**) MDA-MB-231 cells were treated with 1 µM or 2 µM of MLN4924 or DMSO as solvent control for 2 h. Cells were lysed and protein expression was analyzed by western blotting. GAPDH is used as a loading control. The graph shows the quantification of the band density of five independent experiments, normalized to GAPDH and relative to DMSO. All values were normalized to DMSO condition. Values represent mean ± SEM.

Although RhoBTB1 and ROCK1 associated with each other, RhoBTB1 depletion did not affect levels of ROCK1 or ROCK2 or its activation (as determined by MLC phosphorylation) (Supplementary Figure S3B). We, therefore, investigated the possibility that ROCK1 phosphorylates RhoBTB1. RhoBTB1 was found to be phosphorylated in cells, and treatment with the ROCK inhibitor H1152 decreased the phosphorylation of RhoBTB1 (Supplementary Figure S3C). To test whether ROCK1 directly phosphorylates RhoBTB1, an *in vitro* kinase assay was performed using recombinant GST-ROCK1^17–535^ or myc-ROCK1^1–727^ expressed in COS7 cells, and full-length myc-RhoBTB1 expressed in mammalian cells. Immunoprecipitated myc-RhoBTB1 was phosphorylated *in vitro* by GST-ROCK1^17–535^ or myc-ROCK1^1–727^. This phosphorylation was specific to ROCK1, because kinase-dead myc-ROCK1^1–727^ (K105A) was not able to phosphorylate RhoBTB1 (Figure 5A).

To investigate the function of RhoBTB1 phosphorylation, we mutated potential phosphorylation sites. ROCK1 is a serine/threonine kinase and phosphorylation of RhoBTB1 has been detected on several serines and threonines (www.phosphosite.org database; accession date: November 2018). Based on five sites detected in high-throughput mass spectrometry experiments on human RhoBTB1, which are also conserved in the mouse RhoBTB1 sequence, five Ser/Thr residues in RhoBTB1 were mutated to Ala (Figure 5B).

We tested the effect of these RhoBTB1 mutations on its association with Cullin3, which is the only known interaction partner for RhoBTB proteins [5], although the function of this interaction has not been established. We confirmed that RhoBTB1 associated with endogenous Cullin3 and that this association is dependent on its BTB domains (Supplementary Figure S3D). RhoBTB1^T483A^ interacted more with Cullin3 whereas RhoBTB1^T398A^ interacted slightly less compared with wild-type RhoBTB1 (Figure 5C). Notably, T398 is in the first BTB domain, and T483 is close to the second BTB domain, consistent with a role for BTB domains in interacting with Cullin3. The other RhoBTB1 mutations did not appear to affect Cullin3 association. We subsequently investigated the effect of mutating multiple sites. RhoBTB1-S3T1, which had four phospho-site mutations (S69A, S480A, T483A and S485A), had increased Cullin3 association compared with wild-type RhoBTB1, similar to RhoBTB1^T483A^ (Figure 5D). The introduction of a fifth mutation (T398A) caused a decrease in the association (Figure 5D), similar to RhoBTB1 containing the single T398A mutation (Figure 5C, lane 5). These results suggest that RhoBTB1 phosphorylation on T483 inhibits whereas T398 phosphorylation promotes RhoBTB1 and Cullin3 interaction. It is also possible that the mutation of Thr to Ala influences RhoBTB1 and Cullin3 interaction. The relative level of phosphorylation at each of these two sites might thereby alter the ability of RhoBTB1 to target proteins to Cullin3-associated ubiquitin ligase complexes. However, ROCK1 is unlikely to be a target of Cullin3 ubiquitin ligases, because treatment of cells with MLN4924, a small molecule that blocks the ligation of NEDD8 to Cullins and consequently inhibits their activation [23], did not alter ROCK1 levels (Figure 5E).

## Discussion

**Figure 6.**
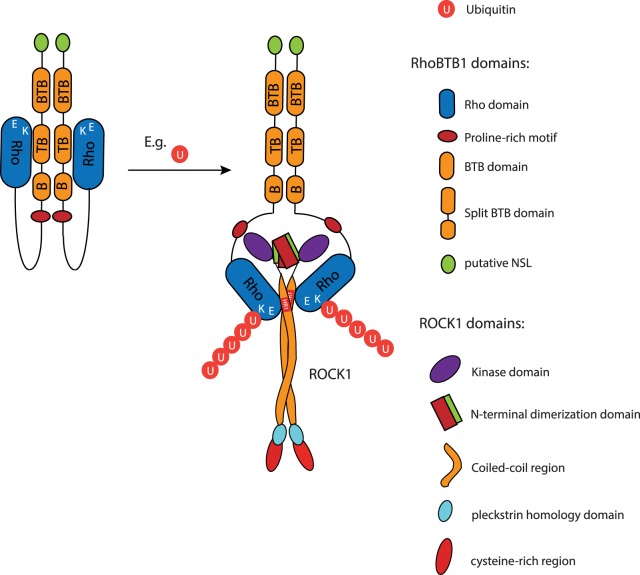
Model for RhoBTB1 interaction with ROCK1. The diagram shows a possible model for the interaction between RhoBTB1 and ROCK1. RhoBTB1 consists of a Rho domain, a PRM, a tandem of 2 BTB domains, of which one of them is split; and a conserved C-terminus with a putative nuclear localization sequence (NLS). We propose that RhoBTB1 is found as a dimer and in a folded conformation. Upon post-transcriptional modification (e.g. polyubiquitination on K197), the Rho domain is released and RhoBTB1 can interact with ROCK1 through its HR1-like domain.

RhoBTB1 is a relatively uncharacterized Rho family member that has not previously been shown to alter cell shape. Here we show for the first time that RhoBTB1 depletion induces PC3 prostate cancer cell elongation in 3D matrices and increases invasion through Matrigel. We previously showed that ROCK1 depletion in PC3 cells leads to cell elongation and increases invasion [17], and indeed our results demonstrate that RhoBTB1 associates with ROCK1 and identify specific regions in each protein involved in this association. ROCK1 is a constitutive homodimer that dimerizes via its long parallel coiled-coil region as well as via the region N-terminal to the kinase domain [11,24]. The region of ROCK1 that is required for association with RhoBTB1 is close to the kinase domain and at the beginning of the coiled-coil region. This region shows some homology to HR1 domains that are found in several RhoA targets, including PKNs, rhotekin and rhophilin [19,21,25,26]. In PKN, the HR1 domain forms an antiparallel coiled-coil structure [26] whereas the equivalent region of ROCK1 and ROCK2 is a long parallel coiled-coil (Figure 6) [24]. This means that RhoA and the Rho domain of RhoBTB1 are likely to interact via a different mechanism with the HR1-like region of ROCK1 compared with RhoA with PKN.

Other proteins have been reported to interact with the parallel coiled-coil domain of ROCK1, including Shroom [27] and RhoA [28]. Interestingly, two molecules of Shroom interact with opposite sides of the parallel coiled-coil [27]. We show here that RhoBTB1 can homodimerize via its BTB domains, similar to RhoBTB2 and RhoBTB3 [5]. It is, therefore, possible that the two Rho domains of the RhoBTB1 dimer associate with either side of the parallel coiled-coil of ROCK1. We hypothesize that, upon post-transcriptional modification (e.g. polyubiquitination on K197), the Rho domain is released and RhoBTB1 can interact with ROCK1 through its HR1-like domain (Figure 6). However, additional experiments would be needed to confirm this model.

It is interesting that a combination of two mutations in different regions of the RhoBTB1 Rho domain act together to increase its association with ROCK1, whereas the individual mutations had no effect. This suggests a model in which these two regions can communicate to reduce ROCK1 binding. Post-translational modification(s) of one or both regions could alter the structure of the Rho domain and/or its interactions with other proteins to enhance ROCK1 binding. For example, K197 is equivalent to K166 in Rac1, which is polyubiquitinated by an SCF family E3 ligase [29].

Our results indicate that RhoBTB1 co-localizes in part with ROCK1 in cells, which is dependent on its ability to associate with ROCK1. In the absence of an active ROCK kinase domain, they both localize to focal adhesion-like structures, which suggests that ROCK1 might alter RhoBTB1 localization. Cells expressing ROCK1 alone show a similar localization for ROCK1 to cells expressing both RhoBTB1 and ROCK1, implying that RhoBTB1 does not alter ROCK1 localization. This contrasts to the effects of Shroom, which interacts with both ROCKs and actin filaments, and localizes ROCKs to specific subcellular locations where it drives actin cytoskeletal changes, and hence is involved in multiple morphogenetic events during development [30,31].

ROCK1, and probably other kinases, phosphorylate RhoBTB1 on multiple sites, promoting or inhibiting its interaction with Cullin3. This could thereby increase or decrease targeting of RhoBTB1-interacting proteins for degradation by Cullin3 ubiquitin ligases. Although ROCK1 associates with RhoBTB1, based on our results it is unlikely to be a target for Cul3-mediated degradation, and thus the identification of other RhoBTB1-binding proteins will be important to determine which proteins are targets for RhoBTB1/Cul3-mediated degradation. Changes in the levels of these proteins could, in turn, contribute to the cell shape changes induced by RhoBTB1 depletion.

The effect of RhoBTB1 depletion on PC3 cell invasion resembles that of ROCK1 depletion, although on 2D substrates RhoBTB1 only has a small effect on cell shape in comparison with ROCK1 [17]. This suggests that RhoBTB1 could act together with ROCK1 predominantly in a 3D environment. In support of our observations on RhoBTB1 in 3D, RhoBTB1 overexpression has recently been reported to reduce invasion of T47D breast cancer cells [32].

In conclusion, we have shown that RhoBTB1 depletion alters cell shape and invasion in 3D matrices, similar to ROCK1/2 depletion. Our results support a model in which RhoBTB1 interacts with ROCK1 to inhibit invasion. In addition, phosphorylation of RhoBTB1 regulates its ability to bind to Cul3, and hence could regulate the targeting of RhoBTB1-associated proteins for Cul3 ubiquitin ligase-mediated degradation.

## Abbreviations

BTB: broad-complex, tramtrack, bric à brac
FCS: fetal calf serum
GAP: GTPase-activating protein
GDI: guanine nucleotide dissociation inhibitor
GEF: guanine nucleotide exchange factor
HRP: horseradish peroxidase
PRM: proline-rich motif
qPCR: quantitative polymerase chain reaction
