# Supplementary material for: RhoBTB1 interacts with ROCKs and inhibits invasion
Source: Biochem J. 2019 Sep 13;476(17):2499–514. doi: 10.1042/BCJ20190203 (PMC6744581; doi:10.1042/BCJ20190203)

Supplementary material

Figure S1. Effects of RhoBTB1 depletion on cell shape and RhoA levels

A) Images showing PC3 cells cultured on plastic or embedded in Matrigel. Scale bar = 100 µm. B) PC3 cells were transfected with siRNA oligo pool targeting RhoBTB1 or siRNA control. After 48 h, cells were embedded in Matrigel. Phase-contrast images were taken after 24 h. C) Quantification of RhoBTB1 mRNA depletion by siRNAs using qPCR and RhoA protein depletion by siRNAs using western blot in PC3 cells. PC3 cells were transfected with siRNA control or siRNA RhoBTB1 oligos #1, #2, #4 or a siRNA pool targeting RhoBTB1 or siRNA RhoA oligos #1, #2. RhoBTB1: after 72 hours, mRNA was isolated and the amount of RhoBTB1 cDNA was determined by quantitative PCR. Graphs show data of three independent experiments. All values were normalized to siRNA control. RhoA: after 72 hours, protein levels were determined by western blot. GAPDH is used as a loading control. Blots are representative of two independent experiments. D) siRNA-transfected PC3 cells were seeded on collagen I-coated coverslips and allowed to adhere and spread for 48 h. Cells were fixed and stained for nuclei (DAPI) and F-actin. Graphs show quantification of 100 cells from three independent experiments using Image J software. Graphs show median, 25th and 75th percentiles and whiskers show the 5th and 95th percentiles. **$p<0.01$, ***$p<0.001$, compared to siRNA control, determined by one-way ANOVA analysis of variance followed by a Dunnett's multiple comparison. Scale bar = 100 µm. (E) RhoA levels after depletion of RhoBTB1. PC3 cells were transfected with siRNA control or siRNA oligos targeting RhoBTB1. After 72 h, cells were lysed and protein expression was analysed by western blotting. GAPDH is used as a loading control. Graph shows the quantification of the band density of three independent experiments.

Figure S2. RhoA and RhoBTB1 association with ROCK1 and conservation of RhoBTB1 between Rho proteins and species

(A) COS7 cells were transfected with pEGFP (empty vector) and vectors encoding myc-ROCK1$^{1\text{-}727}$, myc-ROCK1$^{1\text{-}420}$, GFP-RhoBTB1 and GFP-RhoA^{G14V}. After 24 h, cells were lysed and incubated with GFP-binding protein coupled to agarose (GFP-trap). Total lysates (input) and immunoprecipitates were probed to show levels of myc-ROCK1$^{1\text{-}727}$, myc-ROCK1$^{1\text{-}420}$, GFP-RhoBTB1 and GFP-RhoA^{G14V}. GAPDH is used as a loading control. Blots are representative of three independent experiments. (B) Alignment of all human Rho GTPases. (C) Alignment of the Rho domain of RhoBTB1 in different species. All alignments were performed using T-Coffee and Box-Shade tools (Notredame C, Higgins DG, Heringa J (2000) T-Coffee: A novel method for fast and accurate multiple sequence alignment. *J Mol Biol* **302**, 205-17.; http://sourceforge.net/projects/boxshade/).

Figure S3. RhoBTB1 dimerization, phosphorylation, Cullin3 interaction and effects on ROCK signalling

(A) Top panel: Domain structure of GFP-RhoBTB1 and GFP-RhoBTB1 deletion mutants. RhoBTB1 1-210 = Rho domain; RhoBTB1 1-427 = Rho domain and first BTB domain; RhoBTB1 266-696 = first BTB domain, second BTB domain and C-terminus; RhoBTB1 485-696 = second BTB domain and C-terminus. Bottom panel: COS7 cells were transfected with vectors encoding myc-RhoBTB1, GFP-RhoBTB1 and GFP-RhoBTB1 deletion mutants 24 hours, cells were lysed and incubated with anti-myc-agarose beads. Immunoprecipitates (myc-IP) and total lysates (input) were probed to show levels of myc-RhoBTB1 and GFP-RhoBTB1. Blots are representative of two independent experiments. (B) ROCK1, ROCK2 and p-MLC levels after RhoBTB1 depletion. PC3 cells were transfected with siRNA control and siRNA oligos targeting RhoBTB1. After 72 h, cells were lysed and protein expression was analysed by western blotting. Graph shows the quantification of the band density of three independent experiments. Values represent mean ± SEM. *$p<0.05$, compared to siRNA control, determined by unpaired one-way ANOVA, followed by Dunnett´s test.

(C) COS7 cells were transfected with pRK5-myc (empty vector) or a vector encoding myc-RhoBTB1. After 24 h, cells were treated with 5 µM H1152 (ROCK inhibitor) for 4 h. Cells were then lysed and incubated with anti-myc-agarose beads. Immunoprecipitated lysates were resolved in a SDS-polyacrylamide gel and incubated with Pro-Q Diamond Phosphoprotein Gel Stain. Levels of myc-RhoBTB1 protein were analysed by immunoblotting. Relative levels of phosphorylated myc-RhoBTB1 in H1152-treated cells are shown compared to untreated cells (indicated at bottom). (D) COS7 cells were transfected with vectors encoding GFP-RhoBTB1 and indicated GFP-RhoBTB1 deletion mutants. After 24 hours, cells were lysed and incubated with GFP-binding protein coupled to agarose (GFP-trap). Immunoprecipitates (GFP-IP) and total lysates (input) were probed to show levels of GFP-RhoBTB1 and endogenous Cullin3. Blots are representative of two independent experiments. In all experiments, GAPDH is used as a loading control.

Supplementary Figure 1

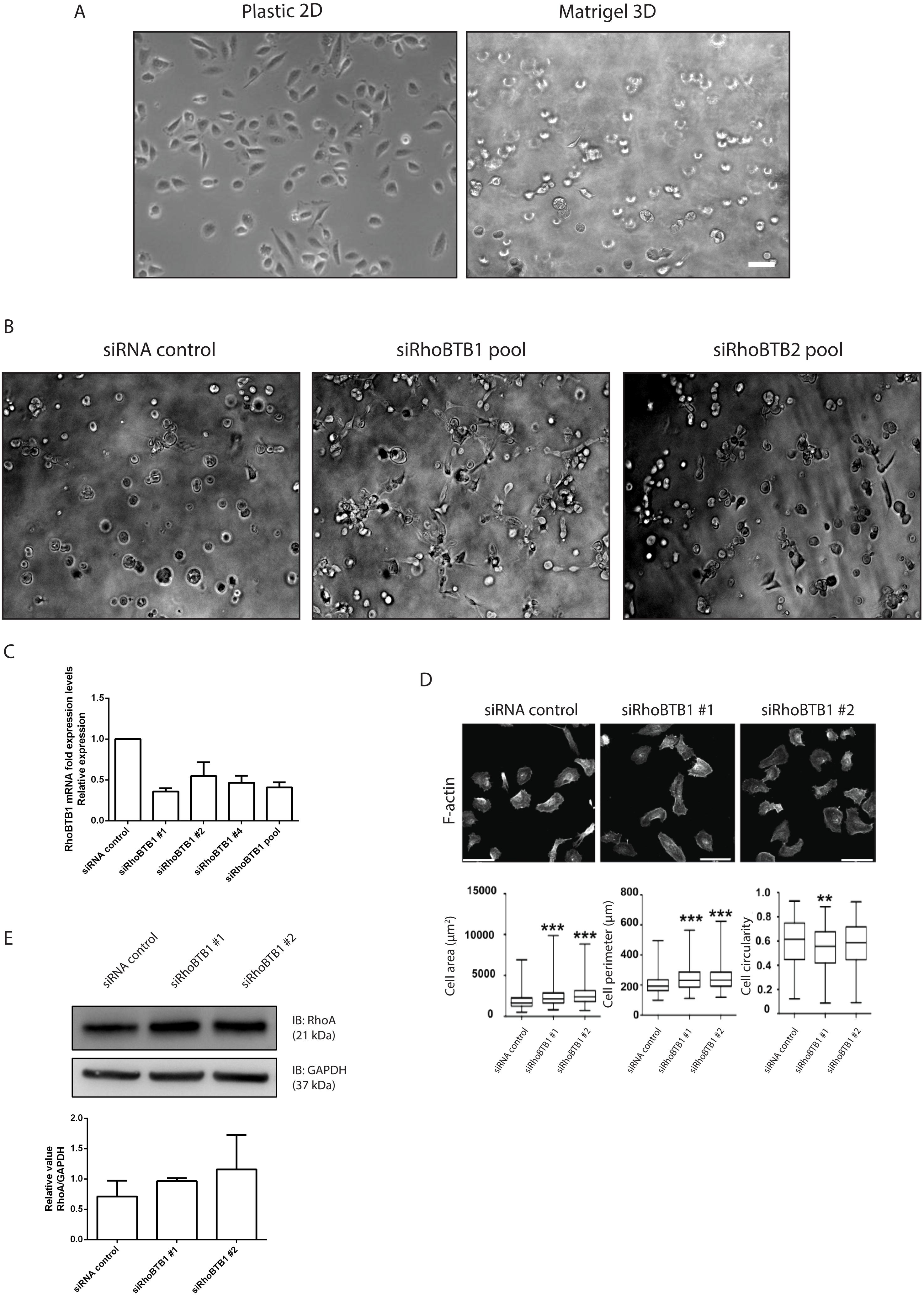

Supplementary Figure 2

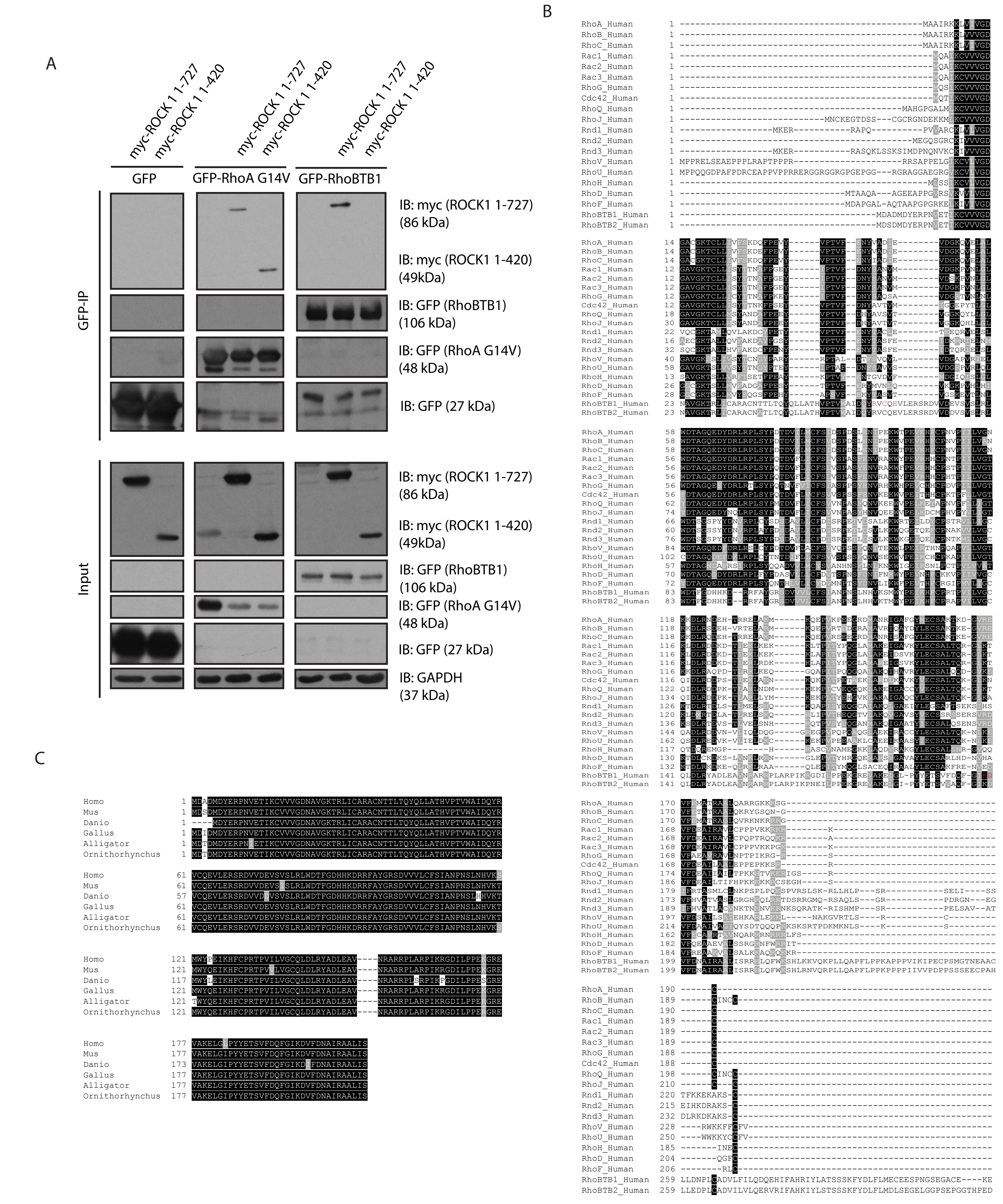

Mutated residues: C60, R62, E64, K197 and D198

Supplementary Figure 3

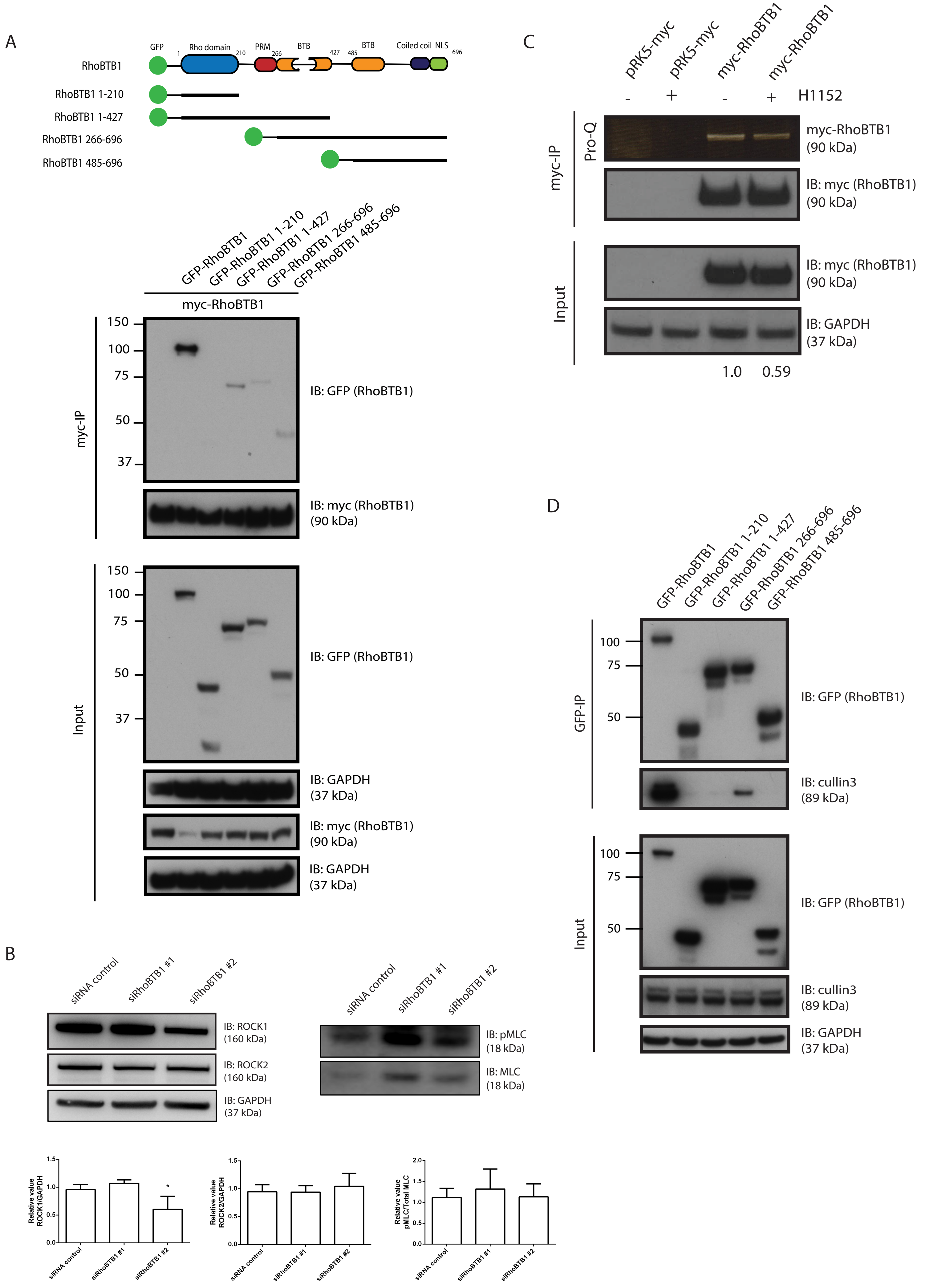

Supplement: Supplementary Figures [file BCJ-476-2499-s1.pdf]
